# Comparative Proteomics of Mulberry Leaves at Different Developmental Stages Identify Novel Proteins Function Related to Photosynthesis

**DOI:** 10.3389/fpls.2021.797631

**Published:** 2021-12-24

**Authors:** Zhiwei Hou, Dashun Xu, Na Deng, Yan Li, Luoling Yang, Shuxuan Li, Hong Zhou, Qintao Huang, Xiling Wang

**Affiliations:** ^1^State Key Laboratory of Silkworm Genome Biology, Southwest University, Chongqing, China; ^2^College of Sericulture, Textile and Biomass Sciences, Southwest University, Chongqing, China

**Keywords:** mulberry, photosynthesis, leaf position, proteome, label-free LC-2MS

## Abstract

Mulberry leaves at different positions are different in photosynthetic rate, nutrient substance and feeding impact to silkworms. Here, we investigated the proteomic differences of the first (L1), sixth (L6), and twentieth (L20) mulberry leaves at different stem positions (from top to the base) using a label-free quantitative proteomics approach. L1 contained less developed photosynthetic apparatus but was more active in protein synthesis. L20 has more channel proteins and oxidoreductases relative to L6. Proteins that detected in all measured leaves were classified into three groups according to their expression patterns in L1, L6, and L20. The protein group that displayed the maximum amount in L6 has the highest possibility that function related to photosynthesis. Nine function unknown proteins belong to this group were further analyzed in the light responsive expression, evolutionary tree and sub-cellular localization analysis. Based on the results, five proteins were suggested to be involved in photosynthesis. Taken together, these results reveal the molecular details of different roles of mulberry leaves at different developmental stages and contribute to the identification of five proteins that might function related to photosynthesis.

## Introduction

Mulberry is a widely distributed deciduous plant with a rapid growth rate. The plant is highly tolerant to abiotic stresses (e.g., drought, high salt content and high temperatures) (Srivastava et al., 2003; Zou et al., 2012; Sajeevan et al., 2017; Gai et al., 2018; Jomngam and Chumpookam, 2018; Liu et al., 2019; Zhang et al., 2020). Mulberry leaves (typically from *Morus alba*) are traditionally used to feed silkworms to produce durable, easy to dye, soft and natural silk fiber that is subsequently used for cloth. The leaves are also used as highly nutritious feed for domesticated animals. The average crude protein content of fresh mulberry leaves ranges from 6.0 to 6.9%, which is substantially higher than that of vegetables (2%) (Gopalan et al., 1971). In addition, mulberry leaves are rich in a wide range of bioactive compounds belonging to steroids, terpenoids, alkaloids and flavonoids (Ma et al., 2014; Li et al., 2020). Proteins involved in the biosynthesis of these compounds await further elucidation.

Photosynthesis, a process that converts light energy into chemical energy stored in carbon skeletons, is the source for plant growth and productivity (Chen et al., 2020). Although significant advances have been made in photosynthesis at the biophysical, biochemical, and molecular levels, photosynthetic complex assembly and regulation still requires vast investigations to better understand the complex and dynamic control mechanisms (Neto et al., 2021). Mulberry is a typical C3 plant. The photosynthesis rate of mature mulberry leaves can reach 40 mg CO_2_/100 cm^2^/h in the fast growing season (Murakami, 1982; Sekhar et al., 2014, 2015). The maximum photosynthesis rate is attained at around 20 days after unfolding, followed by a subsequent decline. During the fast growing season, the 6th leaf (from the top to base) attains the maximum photosynthesis rate, yet in the slow growing season, the maximum photosynthesis rate is only reached at the 30th leaf (Murakami, 1982; Misra et al., 2009).

The leaf area, gas exchange, leaf conductance and saturated net photosynthesis rate are observed to vary with the position of the leaves at the stem. The net photosynthesis rate is relatively low in young and unexpanded leaves and peaks at approximately full-leaf expansion, where it remains stable and subsequently declines (Wilson and Cooper, 1969; Xie and Luo, 2003; Reddy et al., 2017; Gara et al., 2018; Durgadevi and Vijayalakshmi, 2020). The development stage of leaves varies with their positions, therefore different leaf structures (Hu et al., 2020), exhibiting distinct mesophyll cell numbers and chloroplasts per leaf area, resulting in various photosynthetic capacities (Araus et al., 1997; Hu et al., 2020). In addition, leaf position also affects light availability. Aging leaves of forestry trees are typically over-shaded by younger leaves. Light modulates leaf structure. Both the epidermal and mesophyll tissues of light shaded leaves are thinner and the cell density is lower in older leaves due to over-shading (Proietti et al., 2000). Moreover, the leaf composition (e.g., nitrogen content) at different positions can influence photosynthesis. Nitrogen allocation is reported to affect canopy photosynthesis. The younger leaves contain more nitrogen for efficient light utilization and development (Gulmon and Chu, 1981; Kandylis et al., 2009; Piao et al., 2020). However, research on the variations in the photosynthetic apparatus of leaves at different positions in the molecular level is limited. Furthermore, mulberry leaves at different developmental stages are characteristically spirally distributed along the stem. There is currently a lack of studies focusing on the photosynthetic characteristics of mulberry leaves at different positions.

Early estimations of protein profiles in various plant extracts were determined by two-dimensional polyacrylamide gel electrophoresis in plant proteome analysis (Sarma et al., 2008). However, this method is associated with several limitations relating to the membrane protein analysis, the depth analysis, the protein scope, and the identification of small proteins or proteins with an extreme isoelectric point range (Vissers et al., 2007; Rabilloud and Lelong, 2011). In order to overcome these limitations, liquid chromatography-mass spectrometry (LC-MS) technology was applied for the high throughput and quantitative analysis of proteomics. Labeling proteins with stable isotopes such as ^18^O and isobaric tags can potentially increase accuracy (Yao et al., 2001). However, such labeling approaches increase cost, procedural complexity and the potential risk of artifacts (Shen et al., 2009). The quantification of proteins using label-free LC-MS is based on spectral counting or peak area calculations. Subsequently, the use of nano-UPLC allows for the quantitative assessment of changes between samples with high precision yet without the need for stable isotope-based techniques (Silva et al., 2006; Xu et al., 2008). Label-free LC-MS becomes a reliable, low cost and high reproducible method for quantitative proteomics studies (Vanderschuren et al., 2013; Dai et al., 2019; Huihui et al., 2020; Chen et al., 2021).

The protein content of mulberry leaves varies with leaf development, thus resulting in distinct water content, photosynthesis and secondary metabolites levels, and consequently, different feeding characteristics for silkworm. Leaves from the upper region to the base of the stem positions of 6-month-old grafted *Morus alba* branches were morphologically and physiologically different from Leave1 (L1, at the top) to leave20 (L20, at the base). Leave6 (L6) is generally considered to exhibit the highest photosynthetic efficiency amongst all leaves (Murakami, 1982). In order to investigate the protein dynamic profiles and to better understand different roles of mulberry leaves at different developmental stages, we compared the proteomics of mulberry leaves at different positions (L1, L6, and L20) by a label-free LC-2MS method. In particular, we focused on the analysis of protein changes related to photosynthesis and aimed to identify novel proteins that function associated with photosynthesis in mulberry.

## Results

### Mulberry Leaves at Different Developmental Stages Display Various Morphological and Physiological Traits

**FIGURE 1:**
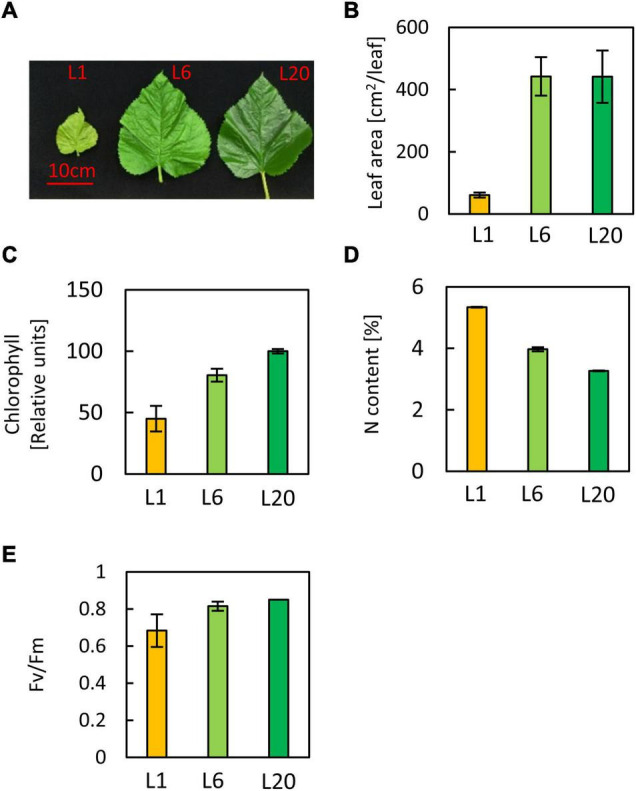
Phenotypes of leaves at different positions of stem. **(A)** Image of mulberry leaves. **(B)** The leaf area of L1, L6, and L20 **(C)** Chlorophyll content of L1, L6, and L20 **(D)** Total nitrogen content and **(E)** the maximum quantum yield of PS? of L1, L6, and L20. Leaves were numbered from top to base of a grafted stem. L1, the first leaf; L6, the sixth leaf, L20, the twentieth leaf.

L1 was paler and smaller than L6 and L20, while L20 showed a similar leaf size but was greener than L6 (Figures 1A,B). The chlorophyll content increased from L1 to L20, in accordance with the phenotype. L6 accumulated 78% more chlorophyll than L1, while L20 contains 44% more chlorophyll than L6 (Figure 1C). Thus, the chlorophyll accumulation rate slows down from L1 to L20. In contrast, the total nitrogen content decreased from L1 to L20 (Figure 1D).

**FIGURE 2:**
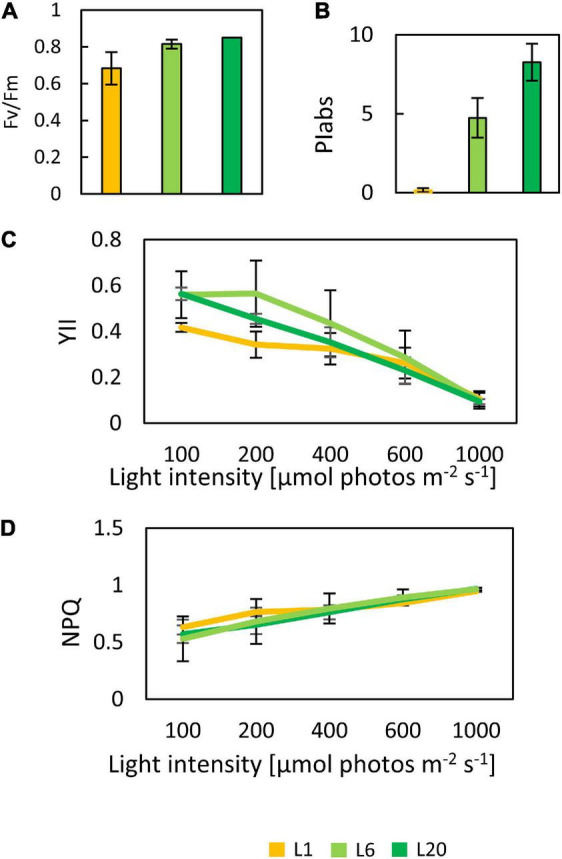
The chlorophyll fluorescence parameter of mulberry leaves at different leaf positions. Fv/Fm **(A)**, PI_ABS_ **(B)**, YII **(C),** and NPQ **(D)** response curves in mulberry leaves at different position 1, 6 and 20 from the top to the base. The date are from three replicated experiments (*n* = 3).

**FIGURE 3:**
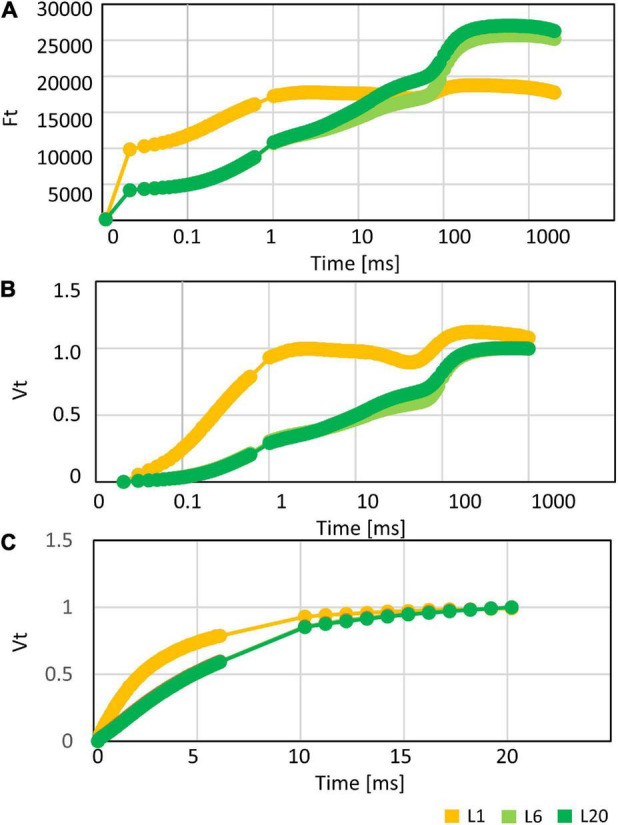
**(A)** The OJIP, **(B)** V_O–P_, and **(C)** V_O–J_ curves of mulberry leaves at position 1, 6, and 20 from the top to the base.

In order to investigate the differences in the photosynthetic capacity of leaves at different positions, we compared the photosynthetic parameters of L1, L6, and L20. The maximum quantum yield of PSII (Fv/Fm) for L1 was only 0.68, while there was no significant difference between L6 and L20 (Figure 2A), indicating increasing PSII activity from L1 to L20. Performance index (PI_ABS_), reflecting the functionality of both PSI and PSII (Strasser et al., 1995; Zivcak et al., 2008), increased from L1 to L20 (Figure 2B). The chemical quantum yield (YII) decreased with the increasing light intensity (Figure 2C). Under moderate light conditions, YII in L1 was significantly lower than L6 and L20, while YII became similar among L1, L6 and L20 when the light intensity was above 600 μmol photos m^–2^ s^–1^ (Figure 2C). In contrast, the non-photo-quenching (NQP) was increased with light intensities, while there was no significant difference among different leaves (Figure 2D). We also compared the electron transfer ability of PSII donor and acceptor by comparing the OJIP curves. The fluorescence intensities at point O, K and J of L1 was much higher than that of L6 and L20 (Figure 3A). However, the maximum fluorescence intensities at point P in L1 was 80% of L6 and L20. The fluorescence intensity at point O was defined as 0 and that at point P or J were defined as 1. Then the relative fluorescence was calculated, resulting in the normalized V_*O*–P_ or V_O–J_ curves. The relative fluorescence in L1 was higher than L6 and L20 from point O to point P (Figure 3B). The increasing magnitude of VJ of L1 was significantly higher than that of L6 and L20 (Figure 3C). No significant difference was observed for OJIP curves in L6 and L20 (Figures 3A–C).

A Coomassie stained gel of proteins separated via SDS-PAGE reveals substantial variation in the overall trends of protein abundance at different molecular weights between L1 and L6 or L20, while values between L6 and L20 were similar (Supplementary Figure 1). L6 and L20 exhibited larger amounts of the RuBisCo large subunit (RBCL) and light harvesting complex (LHC) proteins compared to L1 (Supplementary Figure 1). Most protein bands were strong in L1 and weakened or disappeared in L6 and L20. We then performed a proteomic analysis via a Label-free 2MS method to reveal the protein types and dynamic variations of protein contents in L1, L6, and L20.

### Data Quality Evaluation

We identified a total of 17,107 peptides, with lengths ranging from 7 to 15 amino acid residues (Supplementary Table 1). The measured and theoretical mass-to-charge ratios of the extracted peptide components typically deviated by 5 ppm (parts per million, Supplementary Figure 2A). Most peptides (71.57%) scored more than 60 (Supplementary Figure 2B), while the median score of all peptides was 79 by a program (Andromeda) calculating the match score of peptide-spectrum (Supplementary Figure 2B). These results proved the accuracy and reliability of the identified peptides. In total, 3,048 proteins were detected for all the analyzed leaves (Supplementary Table 2). The repeatability of the detected proteins in triplicate repeats also demonstrates the data quality. Proteins identified in all triplicates accounted for 89.7, 77.5, and 89.4% of all proteins in leaves L1, L6 and L20, respectively. The numbers of proteins identified in more than two runs were 2,569 (L1), 2,144 (L6), and 2,346 (L20), accounting for 96.2, 83.7, and 96.6% of all identified proteins in each group, respectively.

### Classification of Differentially Expressed Proteins

**FIGURE 4:**
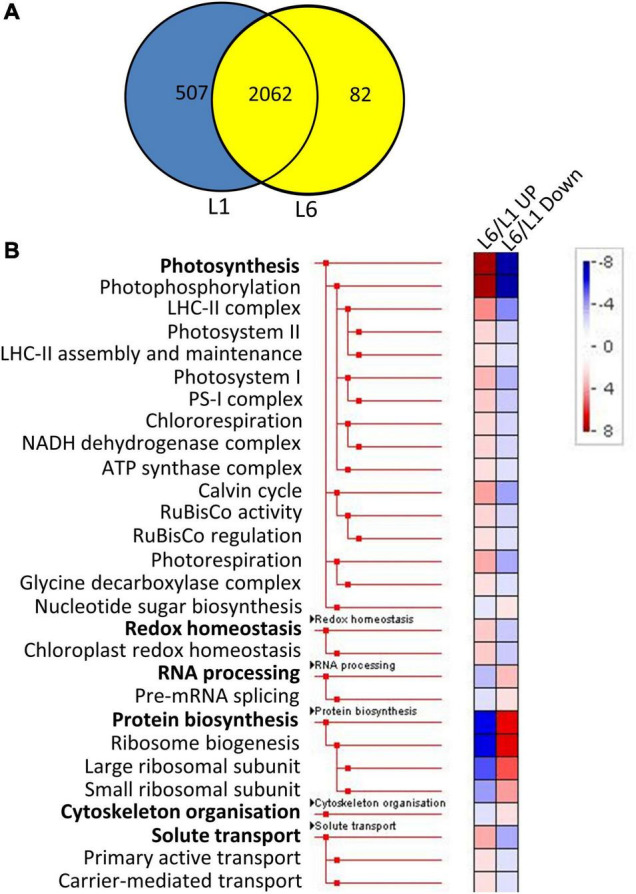
The Venn diagram and functional category enrichment of proteins detected in L1 and L6. **(A)** Shared and unique proteins among L1 and L6. **(B)** Functional category enrichment in L1 and L6. L6/L1 UP, proteins up-regulated in L6 relatively to L1. L6/L1 Down, proteins down-regulated in L6 relatively to L1.

**FIGURE 5:**
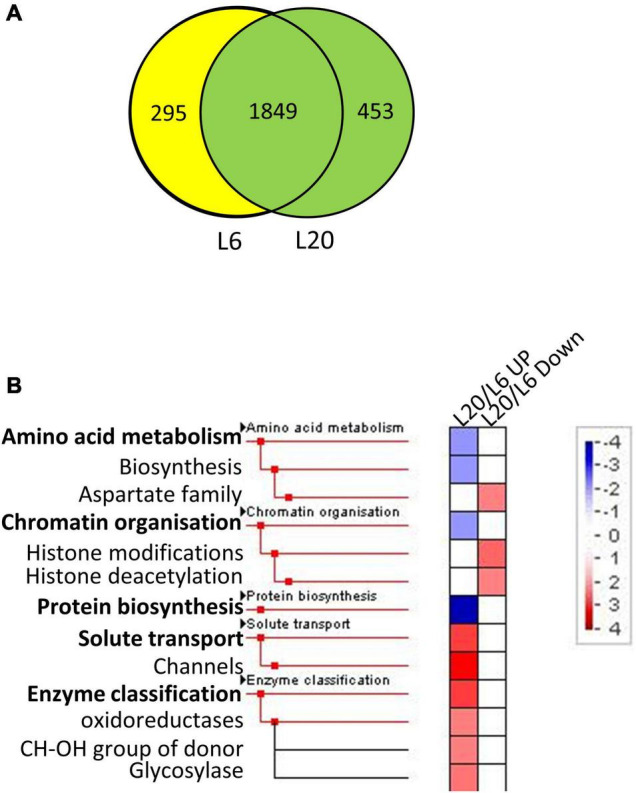
The Venn diagram and functional category enrichment of proteins detected in L6 and L20. **(A)** Shared and unique proteins among L6 and L20. **(B)** Functional category enrichment in L6 and L20. L20/L6 UP indicates proteins showed up-regulated expression in L20 compared to L6. L6/L1 Down indicates proteins showed down-regulated expression in L20 compared to L6.

The proteins identified in at least two runs of triplicate repeats were employed for further bioinformatics analysis. The significance of differentially expressed proteins (DEPs) was specified at 100%. More specifically, a twofold change was employed as a threshold to identify expressions that had experienced a significant up or down regulation for leaves at different developmental stages. Furthermore, a Student’s *t*-test was performed, with *P*-values less than 0.05 indicating a > 95% confidence. A total of 2,062 proteins were detected in both L1 and L6 (Figure 4A). We identified 168 and 249 proteins to be significantly up- and down-regulated in L6 compared to L1, respectively (Supplementary Table 3). DEPs were classified into 36 functional categories using MapMan annotations. Proteins in L6 up-regulated from L1 were significantly over-represented in photosynthesis and less-represented in protein biosynthesis (Figure 4B). In contrast, proteins in L6 that were down-regulated relative to L1 were significantly less-represented in photosynthesis and over-represented in protein biosynthesis (Figure 4B). A total of 1,849 proteins were detected both in L6 and L20, among which 305 exhibited significantly differential expressions in L6 and L20 (Figure 5A). We observed 154 DEPs that increased in L20 compared to L6 (Supplementary Table 4). Those DEPs were enriched in solute transport, particularly for channel proteins and oxidoreductases including the CH-OH donor group and glycosylase. A total of 151 proteins were reduced in L20 compared to L6 (Supplementary Table 4), which were significantly over-represented in the aspartate family, histone modifications and deacetylation (Figure 5B).

**FIGURE 6:**
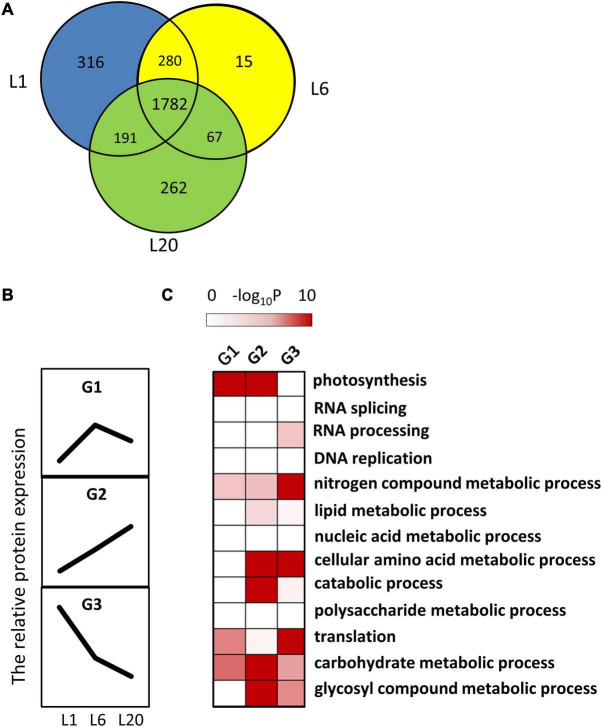
The dynamic progression of leaf proteome from L1 to L20. **(A)** The Venn diagram showed the protein number and percentages of shared or unique proteins among L1, L6 and L20. **(B)** Clustering of the protein amounts of leaf proteome. Three clusters were identified among the 1,782 shared proteins in L1, L6, and L20. **(C)** Functional category enrichment among the clusters.

Moreover, 1,782 proteins were detected in all leaves. These relatively abundant proteins were easily detected in the mulberry leaves (Figure 6A). Among them, 852 proteins were regarded as DEPs that differently expressed in L1, L6, and L20. The DEPs were further grouped by developmental dynamics using the K-Means clustering algorithm as G1, G2, and G3 (Supplementary Table 5). Figure 6B presents the typical expression pattern of each group: G1 proteins exhibited an increased expression in L6 than L1 and a slight decline in L20 compared to L6; G2 proteins were associated with increased protein levels from L1 to L20; and G3 proteins demonstrated a reduction in protein levels from L1 to L20 (Figure 6B). Pathway enrichment testing based on Fisher’s exact test (*P* < 0.01, FDR–5%) reveals proteins involved in photosynthesis to be significantly enriched in G1 and G2, namely those proteins with significant increased protein levels in L6 relatively to L1 (Figure 6C). With the exception of photosynthesis, proteins involved in cellular amino acid metabolic catabolic, carbohydrate metabolic and glycosyl compound metabolic processes were also significantly over-represented in G2. G3 proteins were enriched in nitrogen compound metabolic, cellular amino acid metabolic and translation processes.

**FIGURE 7:**
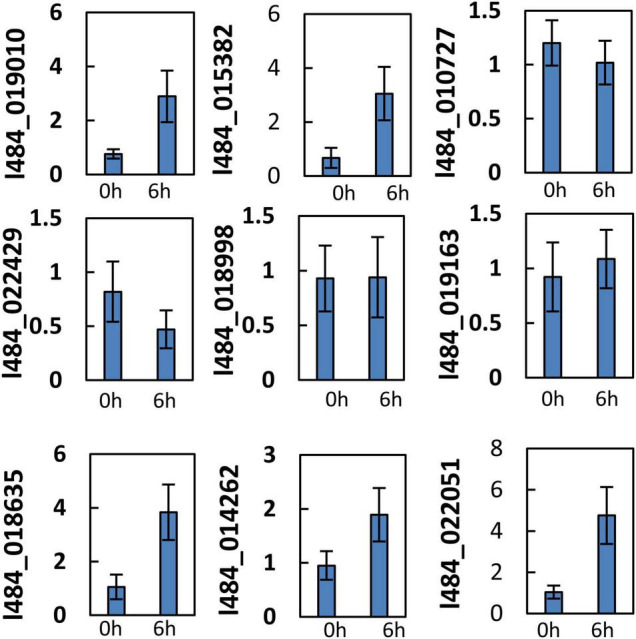
Gene expression of function unknown/uncharacterized genes during greening/deetiolation. Ten-day-old etiolated etiolated seedling of mulberry were exposed to light (100 μmol m^–2^ s^–1^) and harvested before (0 h) or after 6 h′ illumination (6 h) for RNA extraction. The relative gene expression was calculated in relation to β*-ACTIN* (GenBank:HQ163774) by –ΔΔCt method. Error bars indicate mean ± S.E. obtained from three biological repeats.

**FIGURE 8:**
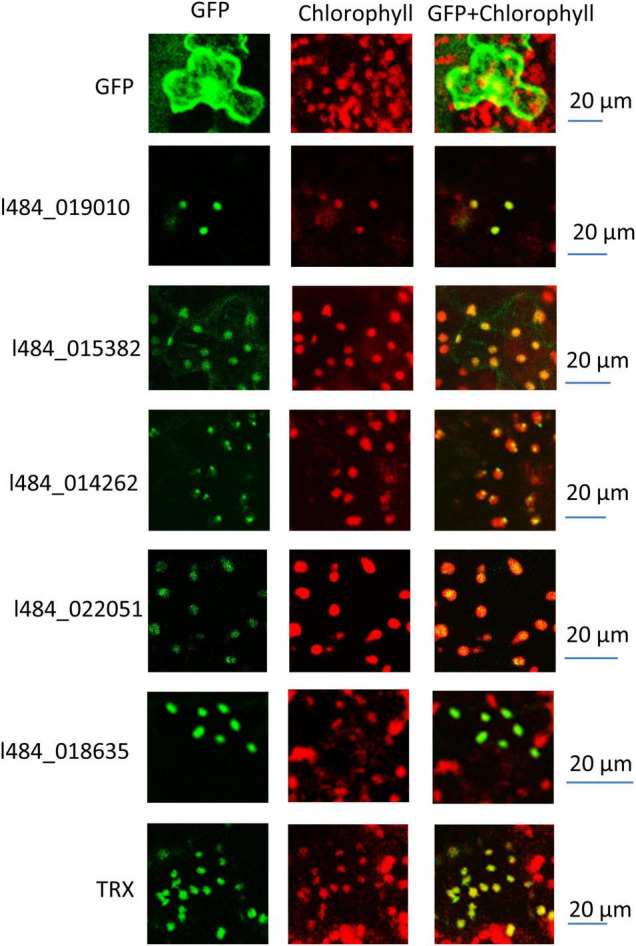
Subcellular localization of function unknown genes. The fused protein with function unknown proteins at the N-terminus and a GFP tag at the C-terminus were transiently expressed in Tobacco leaves. The GFP fluorescence was recorded by a laser scanning confocal microscope. The red fluorescence represents chlorophyll fluorescence and the green fluorescence represents the GFP fluorescence.

G1 contained 32 out of the 105 proteins (30%) with functions related to photosynthesis, while G2 contained 49 out of the total 345 (14%). G1 proteins were likely to provide functions related to photosynthesis, while 9 proteins were associated with unknown functions. We assumed the functions of these proteins likely to be related to photosynthesis. The light-induced greening of the etiolated seedlings is often implemented to investigate the mechanism of light regulation. The expressions of key genes involved in chlorophyll biosynthesis and photosynthesis have been reported to be up-regulated during the onset of greening (Shen et al., 2009; Czarnecki et al., 2011). To verify the relationship of these unknown functions with photosynthesis, the gene expressions of their encoding genes were detected in the etiolated mulberry seedlings during greening. The expressions of 5 out of the 9 total genes were observed to significantly increase following 6 h of illumination in the de-etiolated seedlings (Figure 7). Bioinformatic analysis revealed the homologs of these 5 proteins in the photosynthetic organisms and not in non-photosynthetic organisms (e.g., *E. coli*, yeast and animals). Most proteins exhibited a very low similarity (<35%) in photosynthetic uni-cellular organisms (e.g., *Chlamydomonas reinhardtii*) compared to the mulberry sequences. The l484_022051 encoding-like proteins were not presented in *Physcomitrium patens*. The neighbor-joining phylogenetic trees were constructed for independent proteins using the corresponding homologous protein sequences from *Chlamydomonas reinhardtii*, *Physcomitrium patens*, *Zea mays*, *Oryza sativa*, *Fragaria vesca*, *Populus trichocarpa*, *Daucus carota*, *Arabidopsis*, and *Morus notabilis* (Supplementary Figures 3–7). A close relationship was observed between the proteins in *Morus notabilis* and the homologous proteins in *Fragaria vesca*. Note that *Arabidopsis* and *Zea mays* consistently contained several copies of homologs but only one copy was detected in *Morus notabilis*. To further explore the function of these 5 genes, their subcellular localizations were analyzed by the transient expression of GFP-tagged proteins into tobacco leaves. A thioredoxin protein (TRX) previously reported in the chloroplast was employed as the positive control and the GFP was expressed as the negative control. The five fused proteins was detected in the chloroplasts (Figure 8).

### Protein Abundance of the Photosynthetic Apparatus in L1, L6, and L20, Representing Leaves at Different Developmental Stages

**FIGURE 9:**
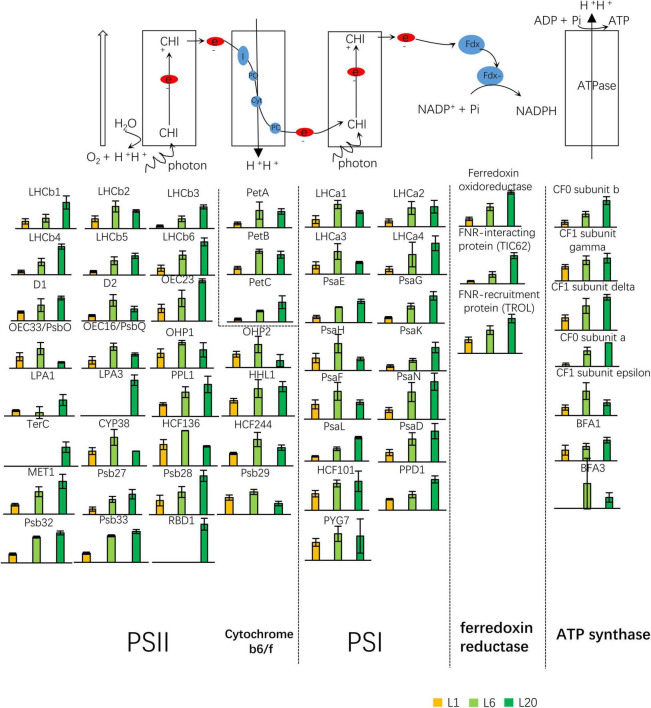
Differential expressed proteins involved in the light reaction of photosynthesis. Twenty eight proteins were the subunit of PSII or involved in the PSII complex assembly or regulation. Three proteins were identified as the subunits of cytochrome b_6_f complex. Fifteen proteins were functional related to PSI complex, three related to ferredoxin reductase and seven related to ATP synthase. PQ, plastoquinone; PQH2, plastoquinol; FNR, Fd-NADP+; PC, plastocyanin.

PSI, PSII, cytochrome b6f complex and ATP synthase are the 4 major complexes involved in the photochemical reaction during photosynthesis. A total of 56 proteins were identified as the components or the assembly proteins involved in photosynthetic electron transport: 30 for PSII, including Oxygen Evolving Complex (OEC) associated to PSII; 3 for cytochrome b6/f complex; 15 for PSI; 3 for ferredoxin oxidoreductase; and 7 for ATPase. Figure 5 depicts the relative expression levels in L1, L6, and L20. The great majority of these proteins exhibited higher protein abundance in L6 compared to L1, while their expressions were inconsistent in L6 and L20. Numerous proteins, such as proteins involved in ferredoxin oxidoreductase were observed to increase in L20 compared to L6, while the opposite was true for OEC33, OEC16, etc. Interestingly, the 3 proteins (RBD1, TerC, and LPA3) involved in the D1 repair cycle were only detected in L20 (Figure 9).

**FIGURE 10:**
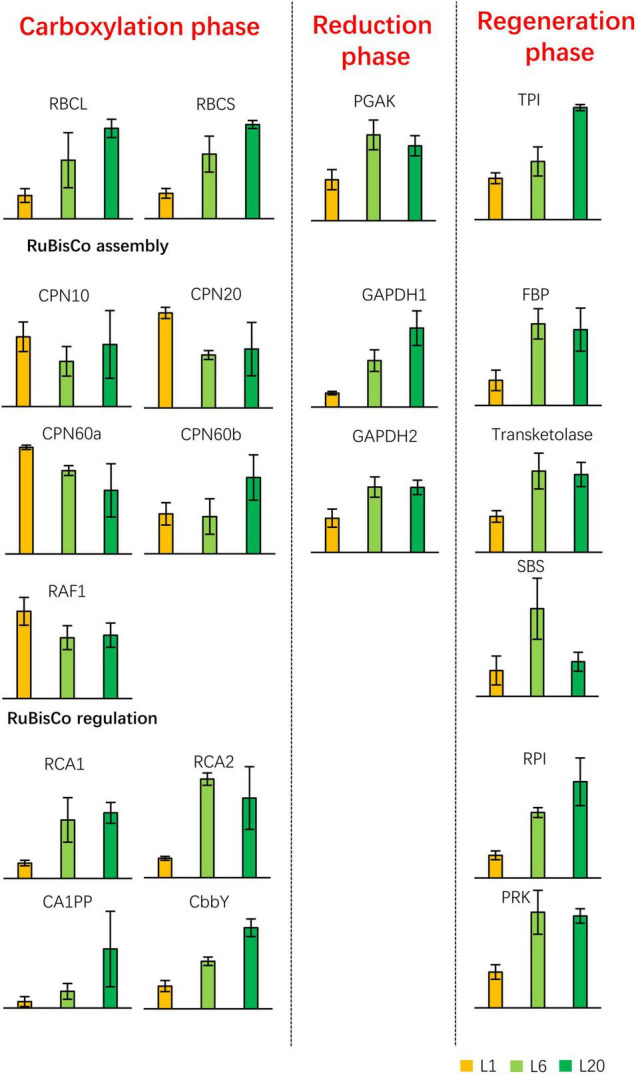
Differential expressed proteins involved in the light-independent reaction of photosynthesis. The Calvin cycle is depicted in the left schematic diagram. PGAK, phosphoglycerate kinase; GAPDH, glyceraldehyde 3-phosphate dehydrogenase; TPI, triosephosphate isomerase; FBP, fructose-1,6-bisphosphatase; SBS, sedoheptulose-1,7-bisphosphatase; PRK, phosphoribulokinase; RPI, phosphopentose isomerase.

We subsequently identified proteins involved in the chemical photosynthesis reaction. The protein contents of RBCL and small subunit of RuBisCo (RBCS) increased from L1 to L20, which is consistent with the previous results of the coommassie staining of protein extracts. With the exception of the proteins involved in the RuBisCo assembly, the proteins were enhanced in L6 compared to L1. The majority of the Calvin cycle proteins (apart from RBCL and RBCS) exhibited a similar protein level in L20 compared to L6, with the exception of CA1PP, GAPDH1, TPI, and RPI, which were the most abundant in L20 (Figure 10). Thus, the protein abundance of the photosynthetic apparatus and regulatory proteins varied from L1 to L20, which makes their differential photosynthetic capacities.

### Chlorophyll Biosynthetic Pathway

We identified 10 proteins as the enzymes involved in chlorophyll biosynthesis, the majority of which were most abundant in L1 compared to L6 and L20 (Supplementary Figure 8). Enzymes including glutamate-1-semialdehyde 2,1-aminomutase, delta-aminolevulinic acid dehydratase, magnesium-protoporphyrin IX monomethyl ester cyclase were observed to gradually decrease from L1 to L20, while porphobilinogen deaminase and magnesium-chelatase subunit H amounts increased in L6 and subsequently decrease in L20. Note that L20 contained a significantly higher amount of protoporphyrinogen oxidase compared to L1 and L6.

### Transcriptional Analysis of Gene Encoding Differentially Expressed Proteins

**FIGURE 11:**
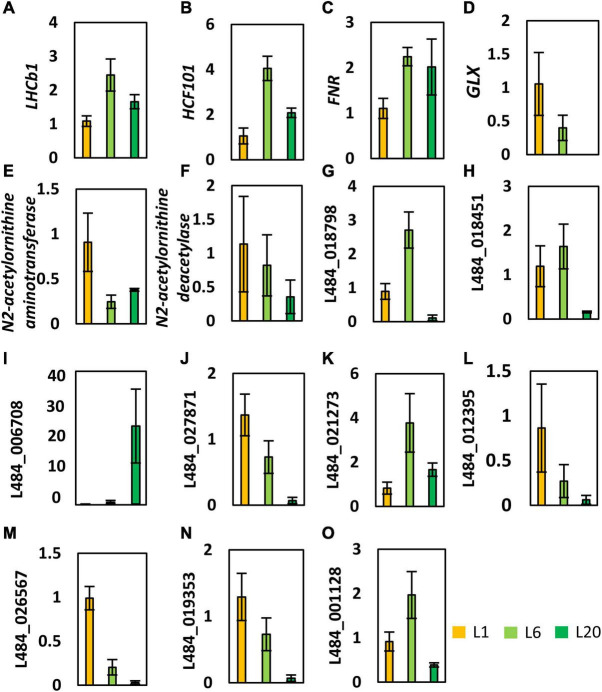
Transcriptional profiles of genes encoding the proteins that showed significantly changed levels in L1, L6, and L20. Gene expression was calculated relative to β-ACTIN (GenBank: HQ163774) using –11Ct method. Error bars indicate average S.E. obtained from three biological repeats. LHCb1 (**A**, L484_020016), HCF101 (**B**, L484_020848) and FNR (**C**, L484_026672) are photosynthesis related genes. GLX (**D**, L484_018049) involved in the cellular respiration process. N2-acetylornithine aminotransferase (**E**, L484_016256) and deacetylase (**F**, L484_023277) involved in the amino acid metabolism. **(G–O)** were randomly picked for transcriptional analysis.

In order to investigate the relationship between the steady-state levels of transcripts and their encoding protein amounts, we performed qRT-PCR analysis on a series of genes that were differentially expressed in L1, L6, and L20. The expression of photosynthesis related genes (*LHCb1, HCF101, and FNR*) were more abundant in L6 compared to L1 (Figures 11A–C). This is in agreement with the corresponding trend in protein levels. However, transcript levels of *LHCb1, HCF101, and FNR* in L6 and L20 leaves are not consistent with their protein levels, indicating that the transcriptional regulation of photosynthesis genes dominates L6 compare to L1, while post-transcriptional regulation plays an important role in L20. A lactoyl-glutathione lyase (GLX1, L484_018049) gene involved in the cellular respiration process exhibited a reduced expression from L1 to L20 (Figure 11D). This is consistent with the corresponding trend in protein levels. Enhanced transcription level expressions of two genes (N2-acetylornithine aminotransferase and deacetylase) involved in the amino acid metabolism were observed in L1 and decreased in L6 and L20 (Figures 11E,F). That is similar to the observed variations in protein abundance. Proteins only detected in 1 or 2 leaves of L1, L6, and L20 were randomly picked for transcriptional analysis (Figures 11G–O). The transcript levels of several genes were consistent with their protein levels (e.g., L484_018798, L484_018451, L484_006708, L484_027871). However, the transcript and protein content of numerous genes varied from L1 to L20 (e.g., L484_021273, L484_012935, L484_026567, L484_019353, L484_001128), indicating the inconsistency in the protein and transcription levels at different leaf positions.

## Discussion

Most DEPs of the first four maize seedling leaves are related to photosynthesis, demonstrating a transition from heterotroph to autotroph from leave1 to leave4 (Chen et al., 2016). Here, we analyzed the proteomics of mulberry leaves at different positions. With the improvement of the proteomic technique, significantly more proteins were identified in L1, L6, and L20, representing leaves at different developmental stages. Compared to L6, L1 contains less photosynthetic apparatus yet a greater amount of proteins involved in the primary metabolisms, particularly protein synthesis, which makes L1 a heterotrophic leaf that consumes energy to enhance growth (Figure 6). L6 is an autotrophic leaf with fully developed photosynthetic apparatus, while L20 contains more proteins that are involved in secondary metabolisms (e.g., oxidoreductases, Figures 4, 5). This may strengthen the adaptability of plant cells to bio or abiotic stresses. These results revealed different roles of mulberry leaves at different developmental stages.

Chlorophylls bind to apoproteins and contribute to the correct folding of these proteins, thus playing an essential role in the stabilization of chlorophyll binding proteins (Seiwert et al., 2017). Our results follow on from this, whereby the majority of LHC proteins were observed to increase from L1 to L20 (Figure 9). This is in agreement with the corresponding trend in chlorophyll content (Figure 1C). Despite the increase in total chlorophyll content from L1 to L20, the chlorophyll accumulation rates were reduced, with a maximum at L1 and subsequently decreasing for L6. This is consistent with the down-regulation of the majority of the chlorophyll biosynthesis genes from L1 to L20 (Supplementary Figure 8).

Chlorophyll a fluorescence was extensively used in probing photosynthesis activities of plants, especially under stress conditions (Wen et al., 2006; Bussotti et al., 2007; Javier Cano et al., 2013; Banks, 2017; Kalaji et al., 2017). However, whether the measured photosynthesis activities corelated with the amounts of accumulated photosynthetic proteins remains open. Fv/Fm values of healthy mature angiosperms leaves typically range within 0.80–0.85 (Schreiber et al., 1995). In the current study, the low Fv/Fm value of L1 is attributed to an incomplete development of PSII. For example, the photosynthetic components, LHCb proteins, D1, D2, and OEC33 and OEC16 content were observed to be lower in L1. This may be owing to the lower gene expression in L1 compared to L6 (e.g., the expression level of *LHCb1* is substantially lower in L1 than L6, Figure 11A). Changes in protein levels may also be a result of different leaf anatomy in L1 and L6. A previous work has reported the ultrastructure (e.g., leaf thickness, cell densities, cell size etc.) to vary between new born and mature leaves in several angiosperms (Proietti et al., 2000; Hu et al., 2020). The PI_ABS_ value is another important indicator of PSII photochemical activity. Here, we observed dramatical differences of leaves from L1 to L20. However, proportions of protein levels of most photosynthetic proteins among different leaves were not correlated with that of Fv/Fm nor PI_ABS_ values. On the other hand, although the protein levels of photosynthetic proteins in L6 and L20 were not consistent, their YII, NPQ, and OJIP curves were not significantly different (Figures 2, 3). Considering that the photosynthesis is a complex and dynamic process (Neto et al., 2021), the photosynthetic activity is not only corelated with the accumulated photosynthetic apparatus but also largely depends on the control mechanisms of biophysical or post-translational levels.

Photosynthesis is highly corelated with leaf nitrogen content in plants (Evans, 1989; Sinclair and Horie, 1989; Reich et al., 1991; Leuning et al., 1995; Evans and Clarke, 2019; Mu and Chen, 2021). A positive correlation between leaf nitrogen content and chlorophyll content was also reported in many species (Ntamatungiro et al., 1999; Nageswara Rao et al., 2001; Mauromicale et al., 2006). Therefore, chlorophyll, nitrogen content and photosynthesis are normally highly corelated with each other, which is the theoretical basis of several remote diagnosis and agricultural technical tools (Netto et al., 2005; Croft et al., 2017; Luo et al., 2019). For example, chlorophyll meter is regularly used in agriculture to screen large plant populations for genotypic differences in leaf nitrogen content and photosynthesis. However, our results revealed a reduction in the nitrogen contents from L1 to L20 while the chlorophyll contents was increased from L1 to L20. Leaf nitrogen content is also previously reported to be higher in younger leaves compared to older leaves (Escudero and Mediavilla, 2003), particularly under nitrogen deficient conditions (Vigneau et al., 2011), which supports our results. We suggested that the rule that chlorophyll content positively correlates with nitrogen content (Munoz-Huerta et al., 2013; Evans and Clarke, 2019; Jia et al., 2021) is not suitable for leaves in different developmental stages which has different portion of proteins involved in other processes except for photosynthetic apparatus which is always associated with chlorophyll. For instance, L1 contains high amounts of proteins involved in protein synthesis, RNA processing, cytoskeleton organization which has nothing to do with chlorophyll; L6 contains more proteins including protein synthesis, amino acid metabolism etc.

The identification of new photosynthetic related proteins can increase our understanding of the regulation of photosystems, the biogenesis of thylakoids, the adaption mechanism to environments, etc. (Stöckel and Oelmüller, 2004; Jin et al., 2014; Shen et al., 2017; Weisz et al., 2019). DEPs detected in leaves L1, L6 and L20 were classified into three groups (G1, G2, and G3) according to their expression patterns in L1, L6 and L20, respectively. The protein amount of G1 and G2 proteins are both higher in L6 compared to L1 but G1 proteins are more abundant in L20 than L6, while G2 proteins maintain similar levels in L20 and L6. In contrast, G3 proteins showed decreased protein levels from L1 to L20. Photosynthesis related proteins were greatly enriched in G1 and G2, accounting for 30 and 14% of G1 and G2 proteins, respectively. The results indicate the G1 proteins to most likely be related to photosynthesis. We identified 9 unknown protein functions in this group, five of which exhibited a significant up-regulation of their expression during the greening of etiolated mulberry seedlings. These five proteins were confirmed to be localized in the chloroplasts (Figure 3). In addition, the homologs of these genes were only present in photosynthetic organisms, some of which were even unique in chloroplast-containing plants. These results strongly suggest photosynthesis-related functions. Phylogenetic analysis of the five proteins reveals the mulberry proteins to be closely related to their homologs in *Fragaria vesca*, which also belongs to Rosales. This is in agreement with previous genome evolutionary analysis (He et al., 2013). Interestingly, only single-copy genes were detected among the five proteins in the mulberry genome, yet more copies were observed in the model plant, *Arabidopsis thaliana*. This mulberry genome trait may facilitate reverse genetic approaches, which play a key role in the identification of novel genes involved in photosynthesis. Previous reports suggest that the mulberry genome evolved much faster than other Rosales (He et al., 2013). This may induce the elimination of pseudo- or unessential mulberry genes. Moreover, ploidy levels and heterozygosity are generally very high in mulberry genomes, thus reducing the need for gene backups. It is promising to generate mutants of these five unknown genes in mulberry to further explore their specific functions. However, methods for gene silencing in mulberry are still missing and the efficient gene manipulation system for mulberry is still limited.

## Conclusion

In conclusion, in the current study we provide detailed information on the variations of protein levels in leaves at different stem positions, corresponding to leaves at different developmental stages, through a label-free 2MS proteomic investigation. The results revealed different roles of leaves at different positions, which might be useful for guidance of the harvesting time and methods for mulberry based agriculture. Besides, we observed that the measured photosynthetic activity is not close corelated with the levels of the accumulated photosynthetic proteins, which implies different control mechanisms of photosynthesis in leaves at different developmental stages. Lastly, five proteins with unknown functions were identified via bioinformatics analysis and experimentally verified as photosynthesis related proteins. However, their specific functions await further investigations.

## Materials and Methods

### Plant Material

*Morus alba* (Jialing50) is a mulberry variety generated by hybridization and ploidy breeding. It has vigorous growth, high leaf yield and good leaf quality for not only sericulture but also for raising poultry and livestock (Xv et al., 2021). Jaling50 was grafted and planted in the mulberry fields of the Sangzhiyuan mulberry collection (Southwest University). Leaves from the top to the base of the scion of the Jialing50 plants were numbered from 1 to 20 (L1–L20). L1, L6, and L20 were harvested for proteomic and transcriptional analysis.

The 10-day-old etiolated seedlings of mulberry (Guiyou 62) were harvested and designated as “0 h” (Onoiko et al., 2017; Jin et al., 2021). Seedlings were then exposed to a white light (100 μmol m^–2^ s^–1^) for 6 h (termed “6 h”).

### Chlorophyll and Total Nitrogen Content Measurements

SPAD measurements were performed using a SPAD-502 Chlorophyll Meter to estimate the relative chlorophyll content in leaves. Approximately 0.5 g leaf material was harvested and digested with 10 mL concentrated sulfuric acid using a graphite digestion apparatus (SH420F, Haineng Instruments, China). The Kjeldahl method was applied to determine total nitrogen using an automatic Kieldahl apparatus (K9860, Haineng Instruments, China) (Kirk, 1950).

### Measurements of Photosynthetic Parameters

Chlorophyll a fluorescence transient (OJIP curve) was measured with a handheld chlorophyll fluorometer (FP110, Photon Systems Instruments, Czech Republic). Measurements were performed on the leaves at a distance of around 2 cm to the major leaf vein close to the leaf center. Each experiment was repeated at least 3 times. Leaves were dark-adapted for 20 min prior to measuring. The OJIP curve was assessed under 3,000 μmol⋅m^–2^ s^–1^ pulse red light, and the recording of the fluorescence signals started at 10 μs and stopped at 1 s after the light pulse. The O, J, I, and P points on OJIP curve correspond to the time points 0, 20, 30, and 1,000 ms, respectively. The fluorescence intensity of O point was defined as 0 and of P point as 1 to calculate the relative fluorescence. The OJIP standardized curve was established by using the equations V_*O*–*P*_ = (Ft-Fo)/(Fm-Fo) and V_*O*–*J*_ = (Ft-Fo)/(FJ-Fo). The maximum photochemical efficiency of PSII (Fv/Fm) and the photosynthetic performance index (PI_ABS_) based on the absorbed light energy values obtained by a JIP-test analysis according to Strasserf et al. (1995).

### Proteomic Analysis

Total proteins were extracted from the leaf materials using SDT solution [4%(w/v) SDS, 100 mM Tris/HCl pH7.6, 0.1M DTT]. Protein content was quantified via bicinchoninic acid (BCA) assay and the proteins were then digested by trypsin. Peptides were desalted using C18 Cartridge and dissolved in 0.1% formic acid. Samples were subsequently injected and separated using a nano-HPLC system by a reverse phase column (Thermo Scientific EASY xcolumn, 10 cm, ID75, 3 μm, C18-A2). The column was balanced with 95% solvent A (0.1% water solution of formic acid) and 5% solvent B (0.1% acetonitrile formate aqueous solution), with a 300 nL/min flow rate. A Q-Exactive mass spectrometer was employed to analyze the separated peptides. The raw data was processed by MacxQuant (v1.5.3.17) for protein identification and quantitative analysis.

### Bioinformatics Analysis

Andromeda was used to calculate the scoring of peptide-spectrum matches. MaxQuant (v1.5.3.17) was then employed for the identification of protein and quantitative analysis. Mapman annotation was applied to assign identified proteins to functional categories. Proteins identified in L1, L6, and L20 leaves were grouped by the Euclid clustering algorithm. Pathway enrichment was determined using agriGO v2.0.^1^

### RNA Extraction and Quantitative Real Time PCR

RNA was extracted from the leaf samples and etiolated seedlings using RNAisoPlus (Takara, Japan). Approximately 30 mg leaf material or etiolated seedlings were homogenized in liquid nitrogen and dissolved in 1 mL RNAisoPlus solution. A total 1 mL chloroform was then added, followed by thorough mixing via a vortex. Following centrifugation for 10 min at 14,400 g, the supernatant was transferred into a new tube and mixed with the same volume of isopropanol. RNA was then pelleted by centrifugation for 10 min at 14,400 g and subsequently washed two times with 500 μL 75% ethanol. The RNA sample was then dissolved in 30 μL RNase free ddH_2_O. RNA concentrations were measured with the NanoDrop 2000 (Biorad). Following DNA digestion, 1 μg RNA was reverse transcribed to cDNA using reverse transcriptase (Takara Japan). Quantitative real time PCR assays were performed in triplicate using the Tower G3 (Analytikjena, Germany). The comparative cycle threshold (CT) method was employed to quantify gene expression, with *ACTIN* taken as the reference gene.

### Separation of Total Protein Extracts From Mulberry Leaves

Approximately 30 mg leaf materials were harvested and homogenized in liquid nitrogen. Samples were then dissolved in 2 × Laemmli buffer and boiled for 10 min. Proteins were separated by SDS-polyacrylamide gel electrophoresis (SDS-PAGE) and stained by Coomassie brilliant blue solution.

### Subcellular Localization of Genes With Unknown Functions

Mulberry gene coding sequences in the absence of the stop codon were sub-cloned into the pSuper1300 vector, resulting in the expression of a fused protein that contains the target protein at the N-terminus and a GFP protein at the C-terminus. The expression of the fused protein was driven by the cauliflower mosaic virus (CaMV) 35S promoter. The construct was then transiently introduced into tobacco leaves and GFP fluorescence was detected with a scanning confocal microscope (Olympus, Japan) following 3 days under dark conditions.

## Data Availability Statement

The datasets presented in this study can be found in online repositories. The names of the repository/repositories and accession number(s) can be found in the article/Supplementary Material.

## Author Contributions

ZH and XW designed the experiments. XW and HZ revised the manuscript. ZH, DX, and ND analyzed the proteomic data. ZH and YL performed the photosynthetic experiments. ZH, LY, SL, and QH performed the transcriptional analysis and the sub-cellular organization analysis. ZH wrote the manuscript. XW revised the manuscript. All authors contributed to the article and approved the submitted version.

## Conflict of Interest

The authors declare that the research was conducted in the absence of any commercial or financial relationships that could be construed as a potential conflict of interest.

## Publisher’s Note

All claims expressed in this article are solely those of the authors and do not necessarily represent those of their affiliated organizations, or those of the publisher, the editors and the reviewers. Any product that may be evaluated in this article, or claim that may be made by its manufacturer, is not guaranteed or endorsed by the publisher.

## Footnotes

1
http://systemsbiology.cau.edu.cn/agriGOv2/specises_analysis.php?&SpeciseID=403&latin=Morus_notabilis

## Supplementary Material

Supplementary Figure 1 Separation of proteins extracted from L1, L6, and L20. **(A)** Coomassie stain of a SDS-PA gel separating protein extracts of L1, L6, and L20. Quantification of relative intensity of protein band representing **(B)** RBCL and **(C)** LHC proteins.

Supplementary Figure 2 The assessment of the quality of LC-2MS data. **(A)** The distribution of peptide mass error. Mass error was calculated by the deviation of measured to desired mass-to-charge-ratio. ppm, parts per million. **(B)** Andromeda Score distribution.

Supplementary Figures 3–7 Evolutionary analysis of function unknown genes by Maximum Likelihood method. Supplementary Figure 3 | L484_019010; Supplementary Figure 4 | L484_015382; Supplementary Figure 5 | L484_018635; Supplementary Figure 6 | L484_014262; Supplementary Figure 7 | L484_022051. Tree was constructed from homologous protein sequences found in nine organisms: *Morus notabilis*; *Daucus carota*; *Populus trichocarpa*; *Zea mays*; *Oryza sativa japonica*; *Arabidopsis thaliana*; *Fragaria vesca*; *Physcomitrium patens*; *Chlamydomonas reinhardtii*.

Supplementary Figure 8 The heat map of the relative protein contents of enzymes for chlorophyll biosynthesis in L1, L6, and L20. Protein amounts in L1 were used as a control to calculate relative fold changes in L6 and L20. The map was constructed by using log2 fold changes. ud represents undetected in all 3 replicates or only detected in 1 out of 3 replicates.

Supplementary Table 1 List of identified peptides in L1, L6, and L20 leaves.

Supplementary Table 2 List of identified proteins in L1, L6, and L20 leaves.

Supplementary Table 3 List of differentially expressed proteins in L6 compared to L1. Up-regulated proteins (168) in L6 relatively to L1 were marked red, and down-regulated proteins (249) were marked green.

Supplementary Table 4 List of differentially expressed proteins in L20 compared to L6. Up-regulated proteins (154) in L20 relatively to L6 were marked red, and down-regulated proteins (151) were marked green.

Supplementary Table 5 Clusters of proteins differentially expressed in L1, L6, or L20.
