# Supplementary figures and images for: Comparative Proteomics of Mulberry Leaves at Different Developmental Stages Identify Novel Proteins Function Related to Photosynthesis
Source: Front Plant Sci. 2021 Dec 24;12:797631. doi: 10.3389/fpls.2021.797631 (PMC8739898; doi:10.3389/fpls.2021.797631)

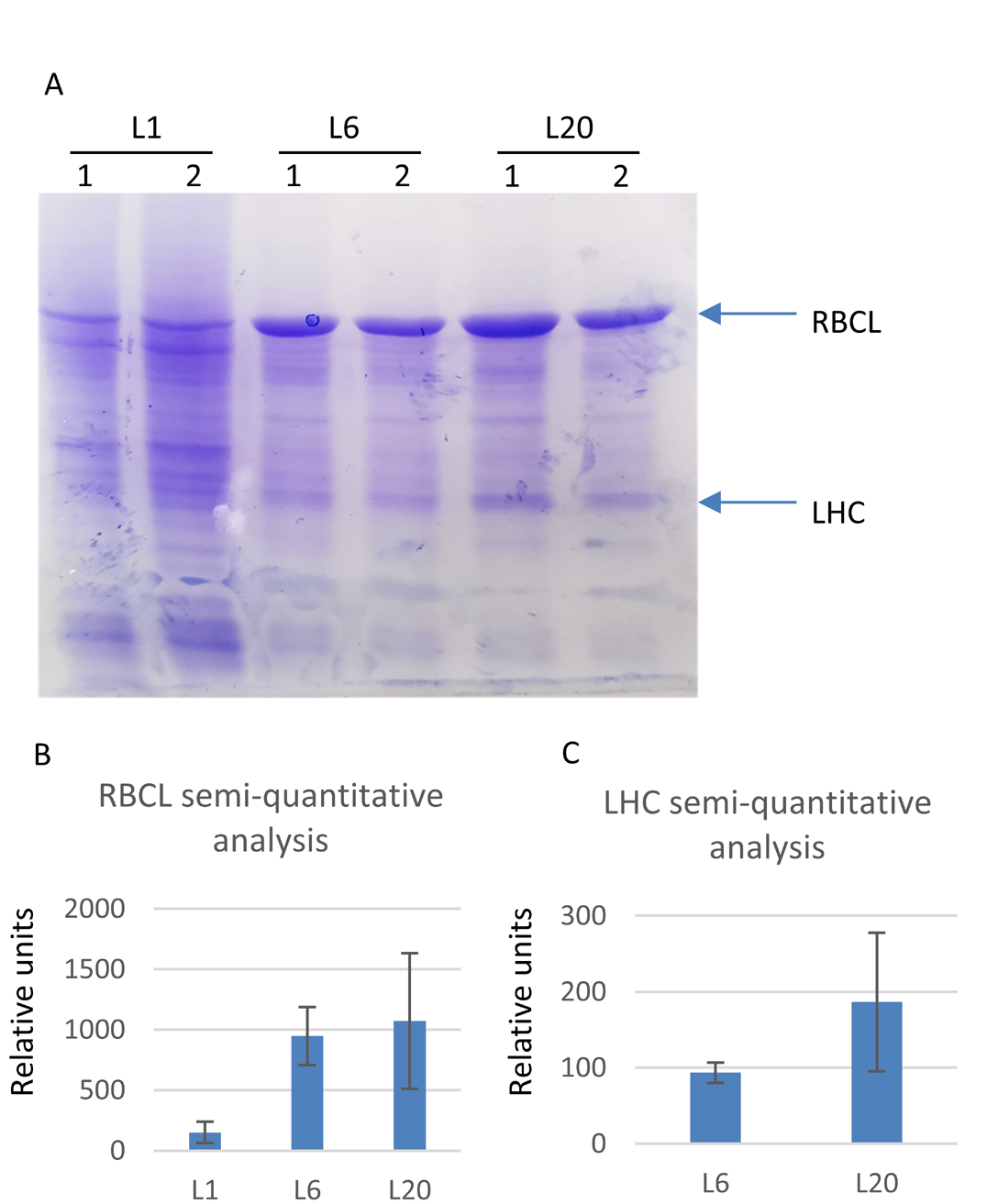

Supplement: Supplementary Figure 1 — Separation of proteins extracted from L1, L6, and L20. (A) Coomassie stain of a SDS-PA gel separating protein extracts of L1, L6, and L20. Quantification of relative intensity of protein band representing (B) RBCL and (C) LHC proteins. [file Image_1.TIF]

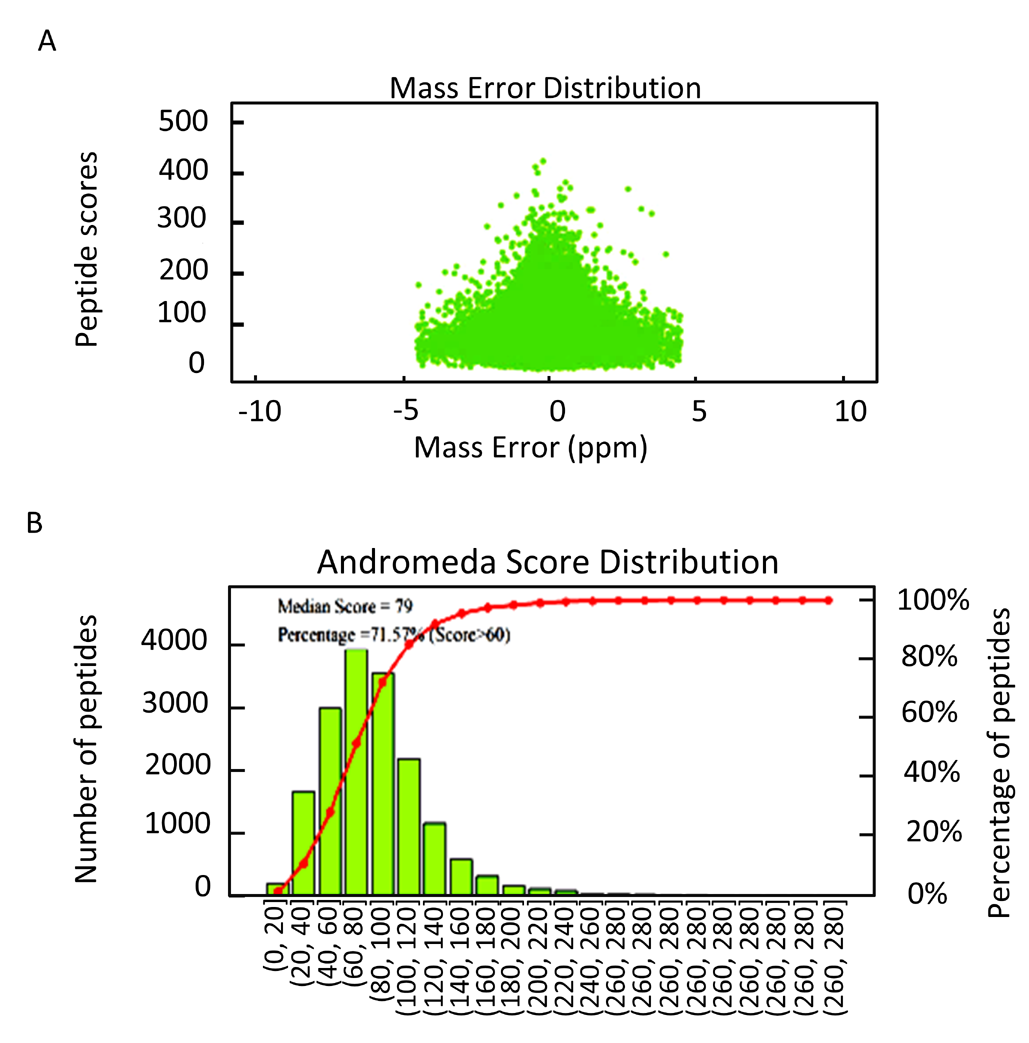

Supplement: Supplementary Figure 2 — The assessment of the quality of LC-2MS data. (A) The distribution of peptide mass error. Mass error was calculated by the deviation of measured to desired mass-to-charge-ratio. ppm, parts per million. (B) Andromeda Score distribution. [file Image_2.TIF]

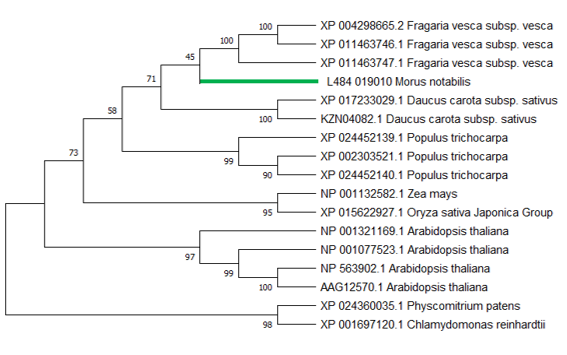

Supplement: Supplementary Figures 3–7 — Evolutionary analysis of function unknown genes by Maximum Likelihood method. Supplementary Figure 3 | L484_019010; Supplementary Figure 4 | L484_015382; Supplementary Figure 5 | L484_018635; Supplementary Figure 6 | L484_014262; Supplementary Figure 7 | L484_022051. Tree was constructed from homologous protein sequences found in nine organisms: Morus notabilis; Daucus carota; Populus trichocarpa; Zea mays; Oryza sativa japonica; Arabidopsis thaliana; Fragaria vesca; Physcomitrium patens; Chlamydomonas reinhardtii. [file Image_3.TIF]

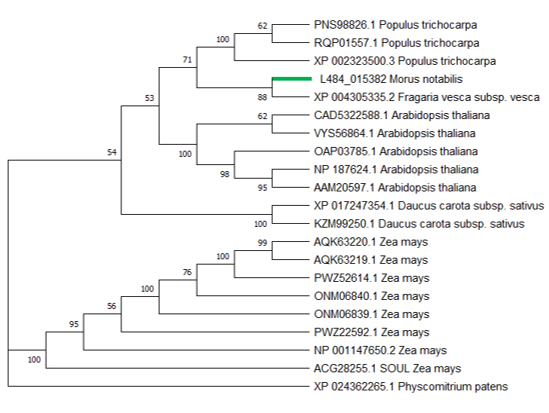

Supplement: Supplementary file 4 [file Image_4.TIF]

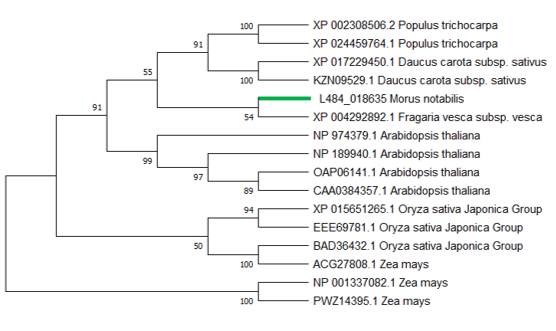

Supplement: Supplementary file 5 [file Image_5.TIF]

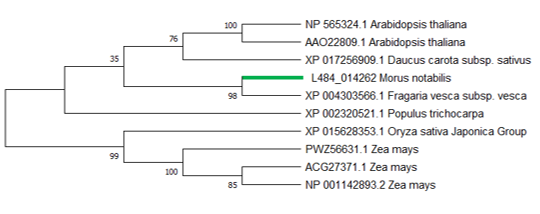

Supplement: Supplementary file 6 [file Image_6.TIF]

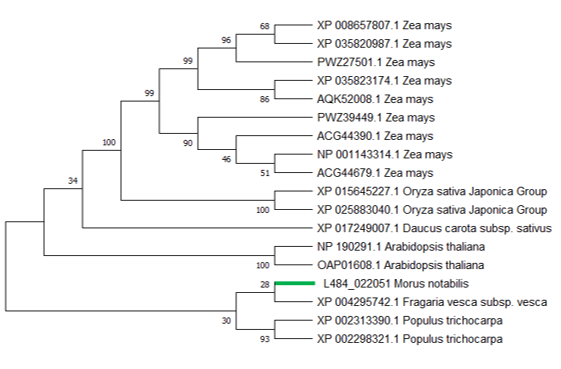

Supplement: Supplementary file 7 [file Image_7.TIF]

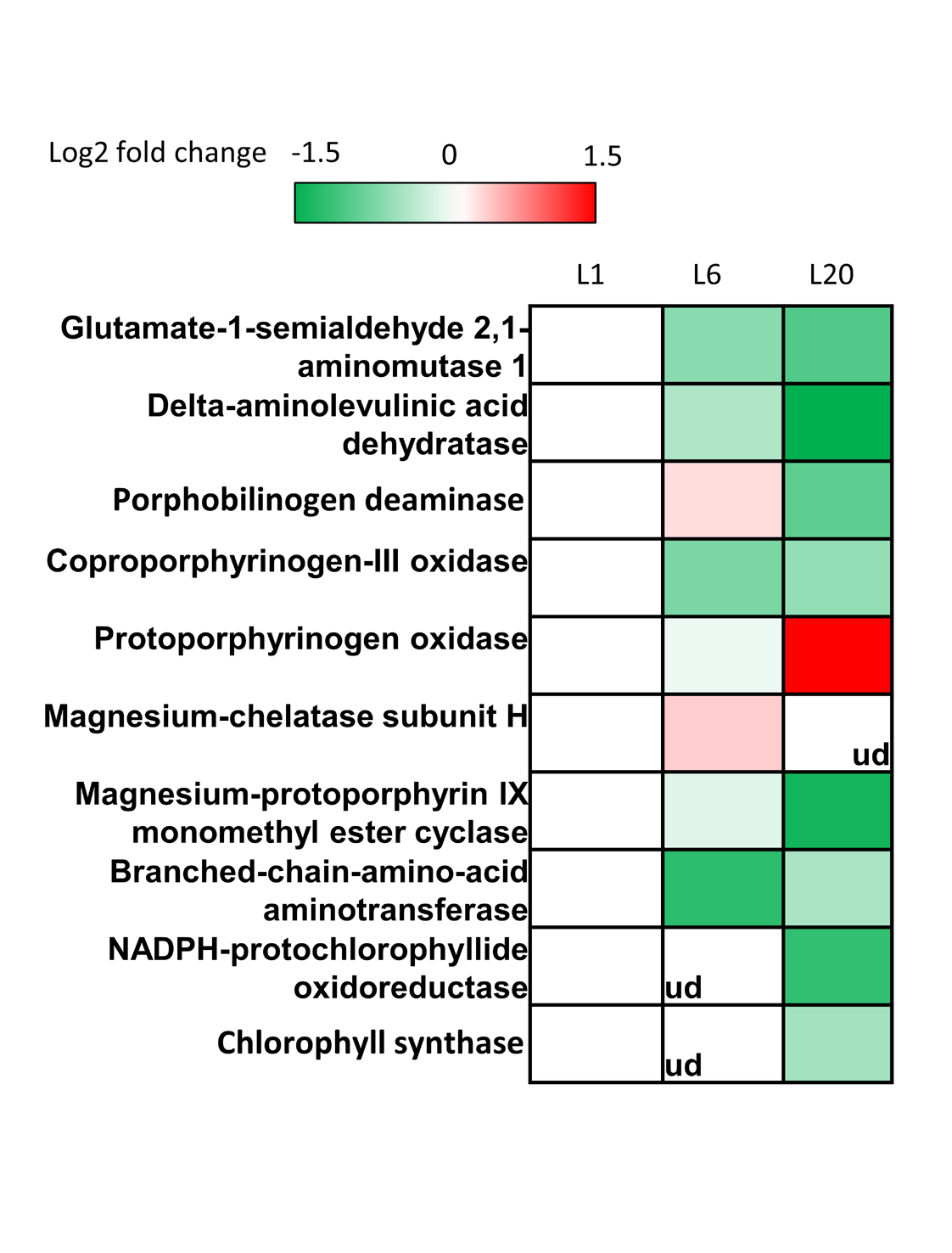

Supplement: Supplementary Figure 8 — The heat map of the relative protein contents of enzymes for chlorophyll biosynthesis in L1, L6, and L20. Protein amounts in L1 were used as a control to calculate relative fold changes in L6 and L20. The map was constructed by using log2 fold changes. ud represents undetected in all 3 replicates or only detected in 1 out of 3 replicates. [file Image_8.TIF]
